# Supplementary material for: Automated feature extraction from population wearable device data identified novel loci associated with sleep and circadian rhythms
Source: PLoS Genet. 2020 Oct 19;16(10):e1009089. doi: 10.1371/journal.pgen.1009089 (PMC7595622; doi:10.1371/journal.pgen.1009089)

S1 Fig. Manhattan plots for genome-wide association studies of sleep and activity traits, derived sleep traits, and circadian traits.

(a) Trait: mean activity levels during sleep

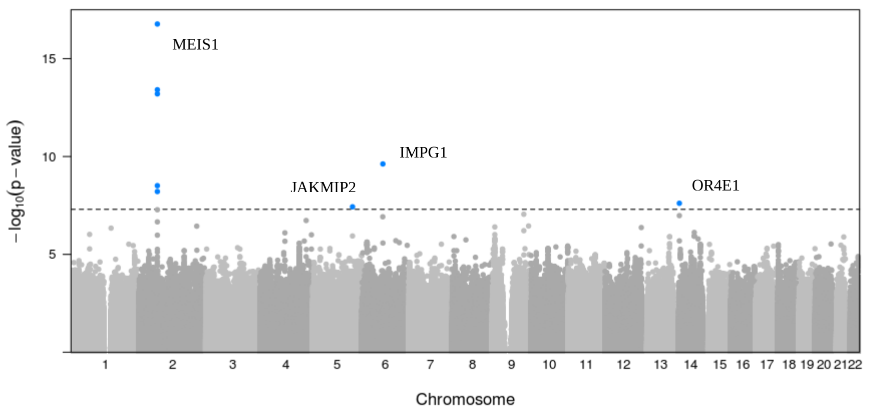

(b) Trait: activity variability during wake

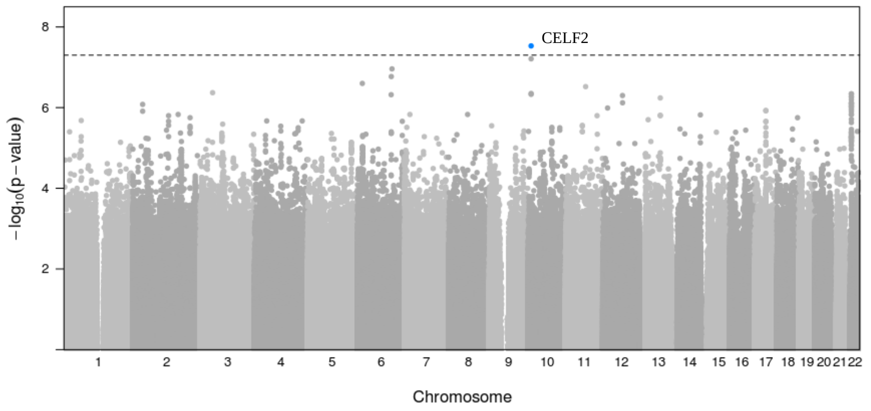

(c) Trait: sleep duration < 5 hours

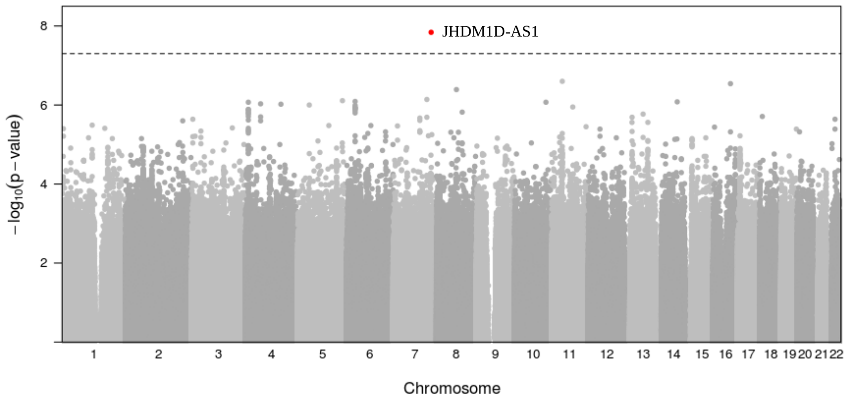

(d) Trait: sleep duration > 10 hours

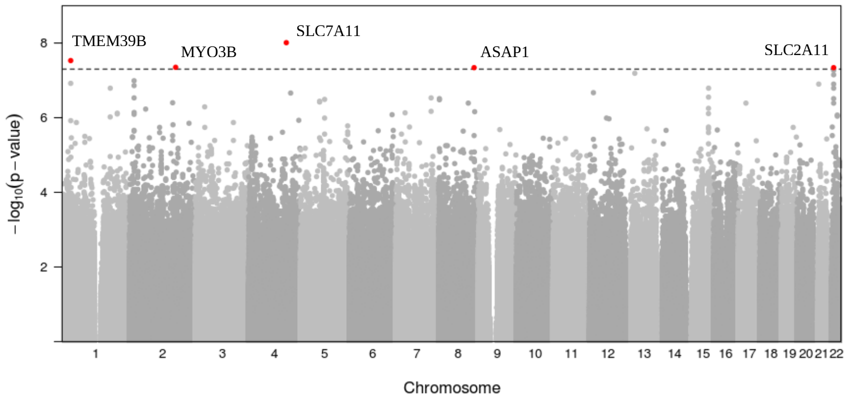

(e) Trait: sleep start time

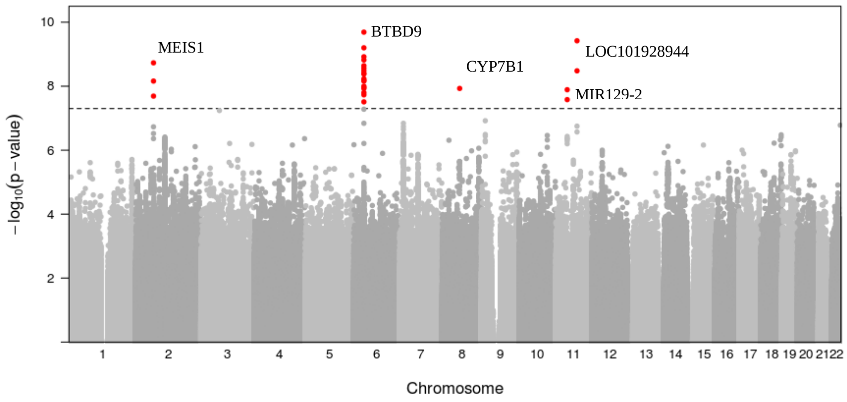

(f) Trait: sleep end time

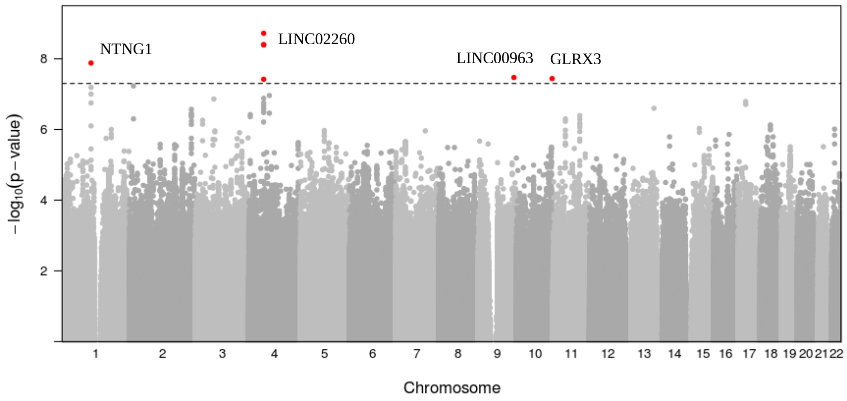

(g) Trait: 1-day periodicity

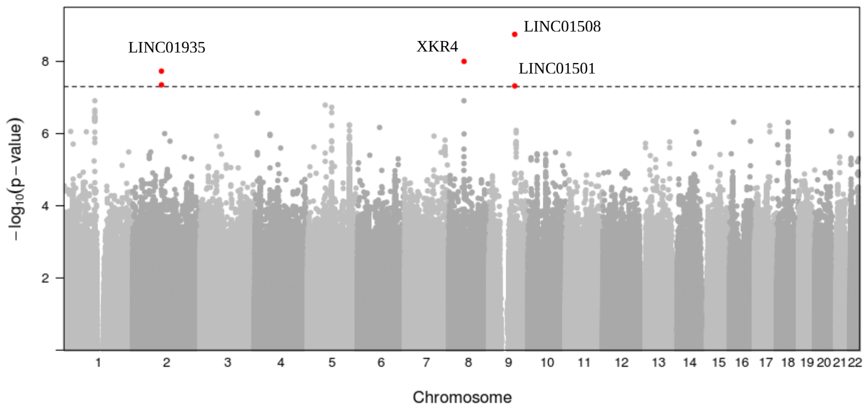

(h) Trait: 1/2-day periodicity

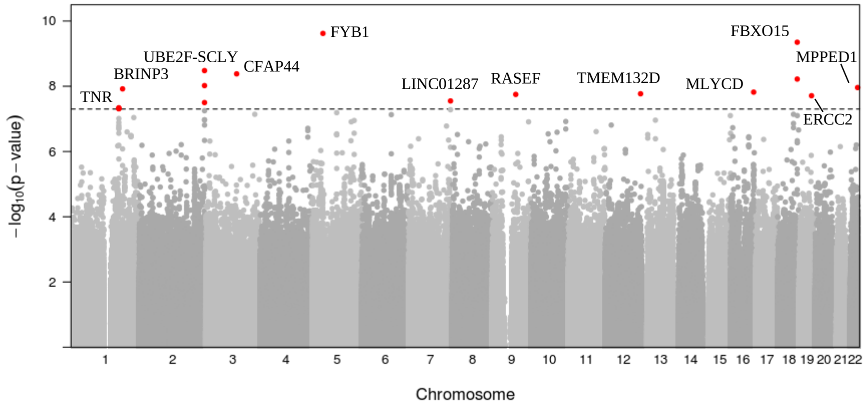

(i) Trait: 1/3-day periodicity

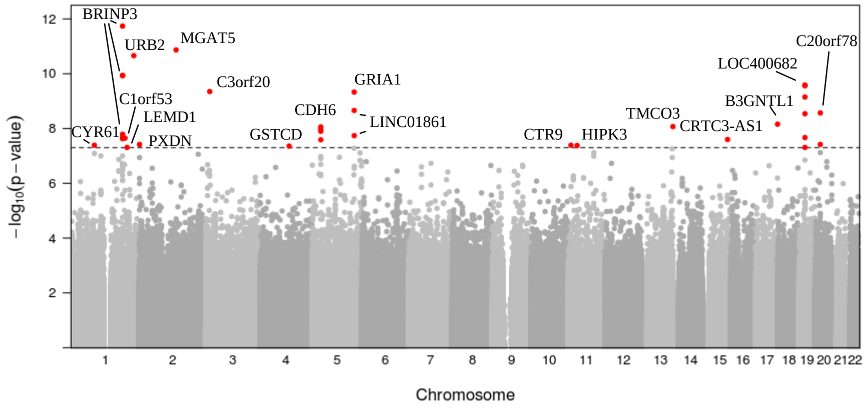

Supplement: S1 Fig — (PDF) [file pgen.1009089.s001.pdf]
