# Supplementary material for: Automated feature extraction from population wearable device data identified novel loci associated with sleep and circadian rhythms
Source: PLoS Genet. 2020 Oct 19;16(10):e1009089. doi: 10.1371/journal.pgen.1009089 (PMC7595622; doi:10.1371/journal.pgen.1009089)
Supplement: S2 Fig — (DOCX) [file pgen.1009089.s002.docx]

S2 Fig. QQ-plots for checking population stratification in genome-wide association studies.

| (a) Mean activity levels during sleep  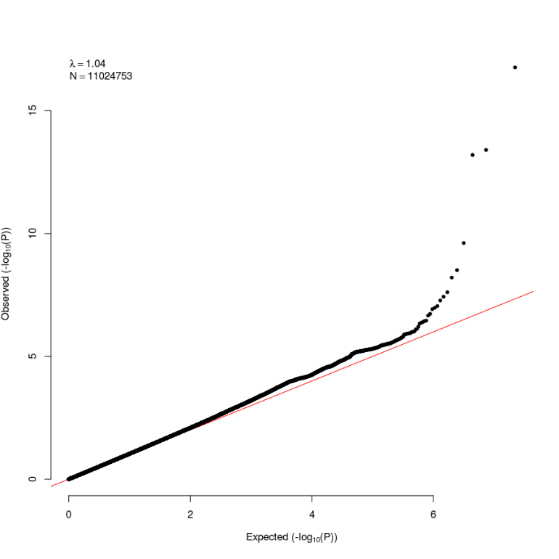 | (b) Activity variability during wake  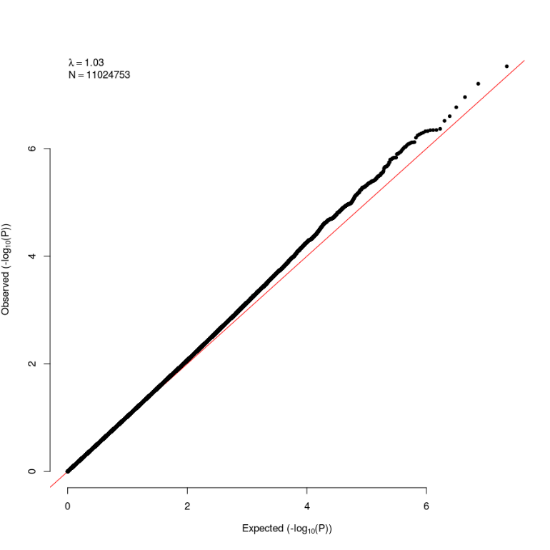 |
| --- | --- |
| (c) Sleep duration < 5 hours  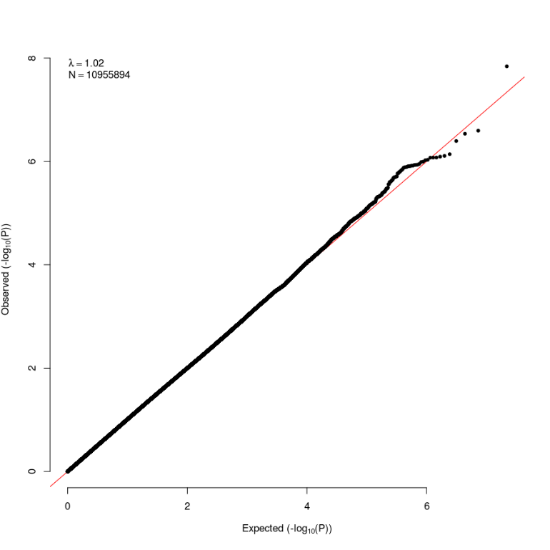 | (d) Sleep duration > 10 hours  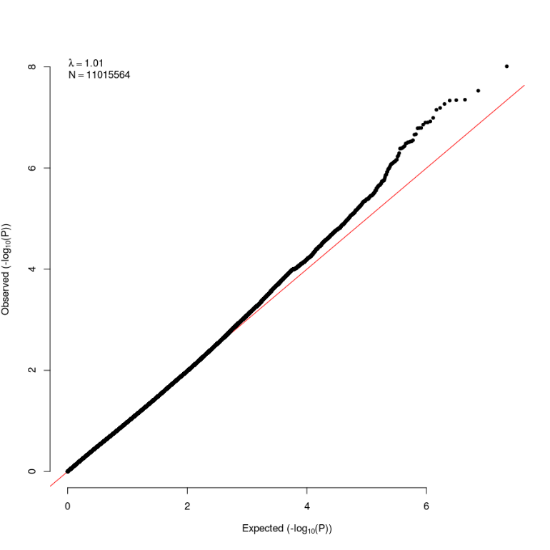 |

| (e) Sleep start  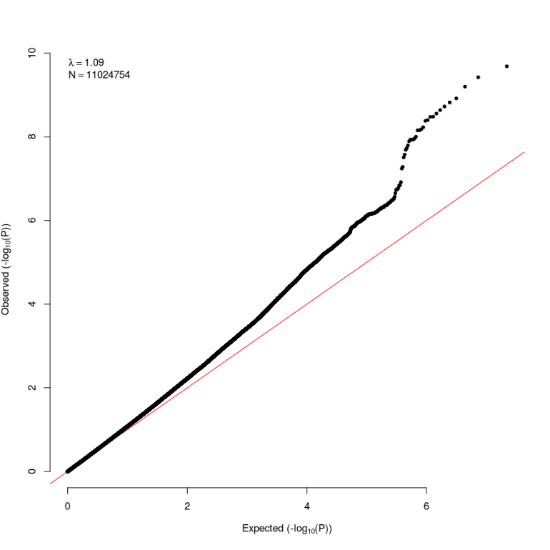  (g) 1-day periodicity  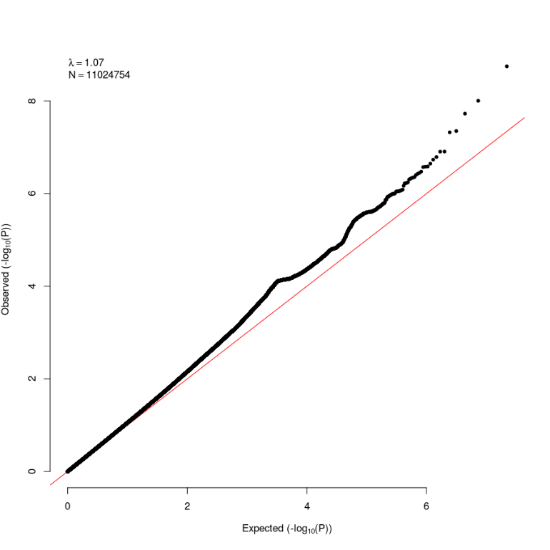  (i) 1/3-day periodicity  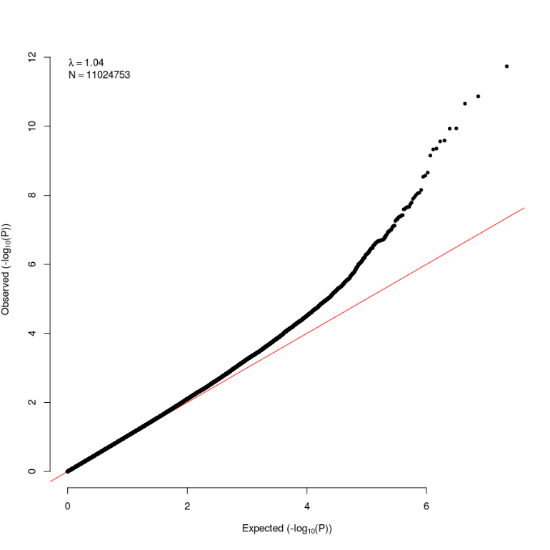 | (f) Sleep end  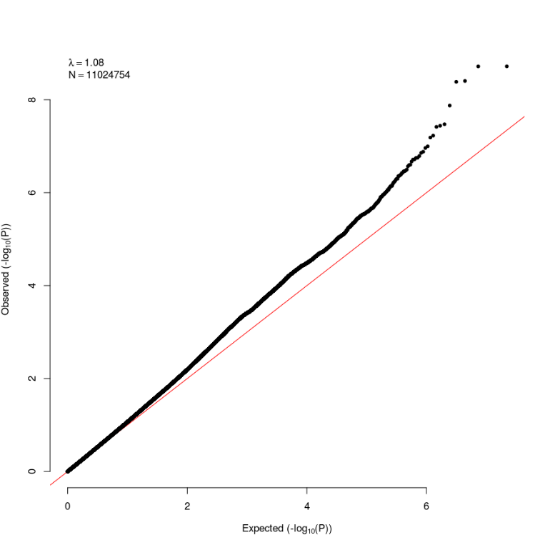  (h) 1/2-day periodicity  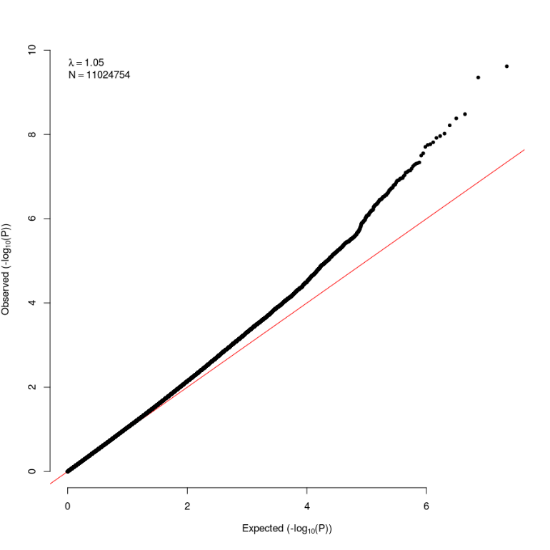 |
| --- | --- |
