# Supplementary material for: Automated feature extraction from population wearable device data identified novel loci associated with sleep and circadian rhythms
Source: PLoS Genet. 2020 Oct 19;16(10):e1009089. doi: 10.1371/journal.pgen.1009089 (PMC7595622; doi:10.1371/journal.pgen.1009089)
Supplement: S1 Table — (DOCX) [file pgen.1009089.s003.docx]

S1 Table. The SNPs identified in genome-wide association studies at the significance level of 5 × 10^−8^ that are associated with sleep start and sleep end traits inferred from accelerometer-measured physical activity in 90,515 UK Biobank participants.

| Trait | Chr | Position | ID | Function | Nearest Gene | Risk Allele | BETA | SE | P |
| --- | --- | --- | --- | --- | --- | --- | --- | --- | --- |
| Sleep Start | 2 | 66750564 | rs113851554 | intronic | MEIS1 | T | 0.070 | 0.012 | 1.88E-09 |
|  | 2 | 66785180 | rs11679120 | intronic | MEIS1 | A | 0.075 | 0.013 | 6.97E-09 |
|  | 2 | 66799986 | rs11693221 | downstream | MEIS1(dist=95) | T | 0.072 | 0.013 | 2.04E-08 |
|  | 6 | 38399009 | rs9349081 | intronic | BTBD9 | A | -0.036 | 0.006 | 1.16E-08 |
|  | 6 | 38401926 | rs78054489 | intronic | BTBD9 | T | -0.036 | 0.006 | 1.10E-08 |
|  | 6 | 38402197 | rs62397049 | intronic | BTBD9 | G | -0.035 | 0.006 | 1.17E-08 |
|  | 6 | 38404140 | rs4714160 | intronic | BTBD9 | T | -0.037 | 0.006 | 3.97E-09 |
|  | 6 | 38404333 | rs9349082 | intronic | BTBD9 | A | -0.037 | 0.006 | 3.32E-09 |
|  | 6 | 38405652 | rs62397050 | intronic | BTBD9 | C | -0.037 | 0.006 | 4.14E-09 |
|  | 6 | 38416590 | rs9380753 | intronic | BTBD9 | A | -0.037 | 0.006 | 2.29E-09 |
|  | 6 | 38418303 | rs4714162 | intronic | BTBD9 | C | -0.037 | 0.006 | 2.77E-09 |
|  | 6 | 38432656 | rs62397055 | intronic | BTBD9 | T | -0.037 | 0.006 | 9.94E-09 |
|  | 6 | 38437303 | rs9369062 | intronic | BTBD9 | C | -0.035 | 0.006 | 6.29E-10 |
|  | 6 | 38438771 | rs4714163 | intronic | BTBD9 | C | -0.035 | 0.006 | 1.51E-09 |
|  | 6 | 38440970 | rs3923809 | intronic | BTBD9 | G | -0.033 | 0.006 | 6.95E-09 |
|  | 6 | 38444040 | rs6920488 | intronic | BTBD9 | G | -0.035 | 0.006 | 1.20E-09 |
|  | 6 | 38447870 | rs13219518 | intronic | BTBD9 | T | -0.037 | 0.006 | 2.06E-10 |
|  | 6 | 38454433 | rs9349087 | intronic | BTBD9 | G | -0.034 | 0.006 | 5.90E-09 |
| Sleep Start | 6 | 38466562 | rs9349088 | intronic | BTBD9 | G | -0.034 | 0.006 | 6.59E-09 |
|  | 6 | 38469187 | rs10947738 | intronic | BTBD9 | C | -0.033 | 0.006 | 1.86E-08 |
|  | 6 | 38469323 | rs10947739 | intronic | BTBD9 | T | -0.033 | 0.006 | 1.60E-08 |
|  | 6 | 38470087 | rs4236060 | intronic | BTBD9 | T | -0.033 | 0.006 | 3.09E-08 |
|  | 8 | 65505208 | rs77576509 | UTR3 | CYP7B1(NM_004820:c.*3991G>C) | C | -0.496 | 0.087 | 1.16E-08 |
|  | 11 | 43671680 | rs79899879 | intergenic | MIR129-2(dist=68647),HSD17B12(dist=30463) | C | 0.470 | 0.084 | 2.65E-08 |
|  | 11 | 43673370 | rs77677460 | intergenic | MIR129-2(dist=70337),HSD17B12(dist=28773) | G | 0.471 | 0.083 | 1.27E-08 |
|  | 11 | 80275391 | rs76322105 | intergenic | LOC101928944(dist=186853) | C | 0.528 | 0.084 | 3.76E-10 |
|  | 11 | 80275893 | rs7109066 | intergenic | LOC101928944(dist=186351) | G | 0.478 | 0.081 | 3.29E-09 |
| Sleep End | 1 | 107738826 | rs60650667 | intronic | NTNG1 | C | 0.512 | 0.090 | 1.33E-08 |
|  | 4 | 55435875 | rs17084481 | intergenic | LINC02260(dist=33503) | T | -0.451 | 0.082 | 3.83E-08 |
|  | 4 | 55442330 | rs17084486 | intergenic | LINC02260(dist=27048) | A | -0.502 | 0.085 | 3.94E-09 |
|  | 4 | 55445652 | rs6833573 | intergenic | LINC02260(dist=23726) | C | -0.515 | 0.086 | 1.91E-09 |
|  | 4 | 55447888 | rs6817676 | intergenic | LINC02260(dist=21490) | T | -0.513 | 0.085 | 1.91E-09 |
|  | 4 | 55467932 | rs114260939 | intergenic | LINC02260(dist=1446) | T | -0.525 | 0.089 | 4.11E-09 |
|  | 9 | 132183853 | rs28361713 | intergenic | LINC00963(dist=67086) | A | -0.508 | 0.092 | 3.37E-08 |
|  | 10 | 132361598 | rs74162666 | intergenic | GLRX3(dist=382952) | A | 0.499 | 0.091 | 3.63E-08 |
