# Supplementary material for: Automated feature extraction from population wearable device data identified novel loci associated with sleep and circadian rhythms
Source: PLoS Genet. 2020 Oct 19;16(10):e1009089. doi: 10.1371/journal.pgen.1009089 (PMC7595622; doi:10.1371/journal.pgen.1009089)
Supplement: S2 Table — (DOCX) [file pgen.1009089.s004.docx]

S2 Table. The SNPs identified in genome-wide association studies at the significance level of 5 × 10^−8^ that are associated with dominant periodicities as circadian traits inferred from accelerometer-measured physical activity in 90,515 UK Biobank participants.

| Trait  (Periodicity) | Chr | Position | ID | Function | Nearest Gene | Risk Allele | BETA | SE | P |
| --- | --- | --- | --- | --- | --- | --- | --- | --- | --- |
| 1-day | 2 | 104066171 | rs144874087 | intergenic | LINC01935(dist=465284) | T | 0.362 | 0.064 | 1.88E-08 |
|  | 2 | 104214869 | rs181820530 | intergenic | LINC01935(dist=613982) | A | 0.345 | 0.063 | 4.46E-08 |
|  | 8 | 56029325 | rs189005747 | intronic | XKR4 | A | -0.148 | 0.026 | 9.90E-09 |
|  | 9 | 93109713 | rs534035399 | ncRNA_intronic | LINC01508 | G | 0.454 | 0.075 | 1.79E-09 |
|  | 9 | 93226520 | rs554696049 | ncRNA_intronic | LINC01501 | C | 0.498 | 0.091 | 4.76E-08 |
| 1/2-day | 1 | 175252640 | rs73046091 | intergenic | TNR(dist=39295) | G | 0.459 | 0.084 | 4.61E-08 |
|  | 1 | 175253053 | rs73046094 | intergenic | TNR(dist=38882) | T | 0.453 | 0.083 | 4.78E-08 |
|  | 1 | 175257794 | rs60264525 | intergenic | TNR(dist=34141) | G | 0.451 | 0.083 | 4.95E-08 |
|  | 1 | 188906152 | rs113154826 | intergenic | BRINP3(dist=1160645) | C | 0.484 | 0.085 | 1.20E-08 |
|  | 2 | 238864452 | rs74387604 | intergenic | UBE2F-SCLY(dist=11135) | C | 0.557 | 0.094 | 3.29E-09 |
|  | 2 | 238868534 | rs77555861 | intergenic | UBE2F-SCLY(dist=7053) | T | 0.541 | 0.094 | 9.52E-09 |
|  | 2 | 238876980 | rs80118729 | ncRNA_intronic | UBE2F-SCLY | G | 0.536 | 0.097 | 3.16E-08 |
|  | 3 | 113025288 | rs75447378 | intronic | CFAP44 | C | 0.544 | 0.093 | 4.15E-09 |
|  | 5 | 39207417 | rs7705606 | intronic | FYB1 | T | 0.523 | 0.083 | 2.41E-10 |
|  | 7 | 152990764 | rs115291013 | intergenic | LINC01287(dist=106240) | G | 0.546 | 0.098 | 2.80E-08 |
|  | 9 | 85674962 | rs182283403 | intronic | RASEF | T | 0.298 | 0.053 | 1.77E-08 |
|  | 12 | 129889219 | rs111638979 | intronic | TMEM132D | G | 0.531 | 0.094 | 1.70E-08 |
|  | 16 | 83928382 | rs116228772 | intergenic | MLYCD(dist=4348) | A | 0.438 | 0.077 | 1.53E-08 |
|  | 18 | 71649540 | rs2850527 | intergenic | FBXO15(dist=91048) | C | 0.511 | 0.088 | 6.07E-09 |
|  | 18 | 71650090 | rs8095104 | intergenic | FBXO15(dist=90498) | T | 0.575 | 0.092 | 4.44E-10 |
|  | 19 | 45859685 | rs3916862 | intronic | ERCC2 | T | 0.483 | 0.086 | 1.96E-08 |
|  | 22 | 43882226 | rs116472104 | intronic | MPPED1 | C | 0.512 | 0.090 | 1.09E-08 |
| 1/3-day | 1 | 86066570 | rs78325101 | intergenic | CYR61(dist=16922) | C | 0.501 | 0.091 | 4.09E-08 |
|  | 1 | 188891995 | rs11811638 | intergenic | BRINP3(dist=1174802) | C | 0.600 | 0.093 | 1.16E-10 |
|  | 1 | 188893474 | rs11800278 | intergenic | BRINP3(dist=1173323) | A | 0.600 | 0.093 | 1.17E-10 |
|  | 1 | 188906152 | rs113154826 | intergenic | BRINP3(dist=1160645) | C | 0.603 | 0.085 | 1.83E-12 |
|  | 1 | 188908979 | rs114764615 | intergenic | BRINP3(dist=1157818) | T | 0.548 | 0.097 | 1.61E-08 |
|  | 1 | 188909688 | rs75413508 | intergenic | BRINP3(dist=1157109) | A | 0.545 | 0.097 | 2.16E-08 |
|  | 1 | 188911763 | rs76933190 | intergenic | BRINP3(dist=1155034) | T | 0.544 | 0.097 | 2.33E-08 |
|  | 1 | 197876533 | rs1546244 | downstream | C1orf53(dist=36) | C | 0.444 | 0.079 | 2.24E-08 |
|  | 1 | 205357143 | rs6686635 | intronic | LEMD1 | G | 0.528 | 0.097 | 4.89E-08 |
|  | 1 | 229830113 | rs572888683 | intergenic | URB2(dist=34166) | T | 0.457 | 0.068 | 2.21E-11 |
|  | 2 | 1609658 | rs73168450 | intergenic | PXDN(dist=26001) | G | 0.431 | 0.078 | 3.77E-08 |
|  | 2 | 135072760 | rs570561789 | intronic | MGAT5 | G | 0.581 | 0.086 | 1.35E-11 |
|  | 3 | 14801742 | rs76226780 | intronic | C3orf20 | C | 0.521 | 0.084 | 4.43E-10 |
|  | 4 | 106783596 | rs188605162 | intergenic | GSTCD(dist=14714) | C | 0.519 | 0.095 | 4.40E-08 |
|  | 5 | 31252054 | rs78428804 | intronic | CDH6 | G | 0.552 | 0.099 | 2.55E-08 |
|  | 5 | 31258987 | rs115523641 | intronic | CDH6 | G | 0.583 | 0.102 | 1.13E-08 |
|  | 5 | 31260648 | rs77152682 | intronic | CDH6 | T | 0.580 | 0.102 | 1.27E-08 |
|  | 5 | 31264101 | rs59775462 | intronic | CDH6 | C | 0.575 | 0.100 | 8.77E-09 |
|  | 5 | 31264615 | rs115687789 | intronic | CDH6 | A | 0.573 | 0.100 | 9.89E-09 |
|  | 5 | 153195847 | rs544388919 | intergenic | GRIA1(dist=2418) | G | 0.438 | 0.070 | 4.67E-10 |
|  | 5 | 153271609 | rs146810346 | ncRNA_intronic | LINC01861 | G | 0.411 | 0.069 | 2.20E-09 |
|  | 5 | 153322873 | rs184603073 | intergenic | LINC01861(dist=44326) | A | 0.399 | 0.071 | 1.81E-08 |
|  | 11 | 10779846 | rs188558150 | intronic | CTR9 | G | 0.364 | 0.066 | 4.03E-08 |
|  | 11 | 33308084 | rs148530680 | exonic | HIPK3 | C | 0.422 | 0.077 | 4.18E-08 |
|  | 13 | 114207113 | rs182147357 | intergenic | TMCO3(dist=2569) | A | 0.454 | 0.079 | 8.43E-09 |
|  | 15 | 91175860 | rs141991588 | ncRNA_intronic | CRTC3-AS1 | A | 0.467 | 0.084 | 2.51E-08 |
|  | 17 | 80972046 | rs182736485 | intronic | B3GNTL1 | T | 0.401 | 0.069 | 6.96E-09 |
|  | 19 | 21812746 | rs7245831 | intergenic | LOC400682(dist=19724) | T | 0.575 | 0.091 | 2.74E-10 |
| 1/3-day | 19 | 21812763 | rs146923867 | intergenic | LOC400682(dist=19707) | C | 0.576 | 0.091 | 2.60E-10 |
|  | 19 | 21815922 | rs113115412 | intergenic | LOC400682(dist=16548) | C | 0.563 | 0.091 | 7.04E-10 |
|  | 19 | 21821327 | rs112450146 | intergenic | LOC400682(dist=11143) | G | 0.598 | 0.101 | 2.90E-09 |
|  | 19 | 21822329 | rs116464036 | intergenic | LOC400682(dist=10141) | G | 0.550 | 0.098 | 2.16E-08 |
|  | 19 | 21822330 | rs115454713 | intergenic | LOC400682(dist=10140) | C | 0.533 | 0.098 | 4.87E-08 |
|  | 20 | 18850460 | rs149624949 | intergenic | C20orf78(dist=39613) | G | 0.372 | 0.063 | 2.68E-09 |
|  | 20 | 18855109 | rs375329986 | intergenic | C20orf78(dist=44262) | A | 0.336 | 0.061 | 3.78E-08 |
