# Supplementary material for: Automated feature extraction from population wearable device data identified novel loci associated with sleep and circadian rhythms
Source: PLoS Genet. 2020 Oct 19;16(10):e1009089. doi: 10.1371/journal.pgen.1009089 (PMC7595622; doi:10.1371/journal.pgen.1009089)
Supplement: S3 Table — (DOCX) [file pgen.1009089.s005.docx]

S3 Table. Estimated genetic correlation of sleep and circadian traits with other complex traits and the corresponding p-values.

|  | | | Genetic Correlation (p-values) | | | | | | | | | | | |
| --- | --- | --- | --- | --- | --- | --- | --- | --- | --- | --- | --- | --- | --- | --- |
| General Category | UKBiobank  Category | Category  Details | Mean Activity During Sleep | Activity  Variability During  Sleep | Mean Activity During Wake | Activity Variability During Wake | Sleep Duration | Sleep Duration  >10h | Sleep Duration <5h | Sleep Start | Sleep End | 1-Day Periodicity | 1/2-Day Periodicity | 1/3-Day Periodicity |
| Physical Activity | 1001 | Duration of strenuous sports | -0.059 (8.30e-01) | -0.438 (1.68e-01) | 0.183 (3.87e-01) | -0.132 (6.43e-01) | -0.070 (7.67e-01) | -0.239 (5.65e-01) | 0.009 (9.79e-01) | 0.025 (9.05e-01) | 0.114 (5.41e-01) | 0.232 (2.32e-01) | -0.380 (1.33e-01) | -0.183 (5.48e-01) |
|  | 104900 | Time spent doing vigorous physical activity | -0.051 (7.65e-01) | -0.166 (2.79e-01) | 0.044 (7.97e-01) | 0.024 (8.90e-01) | 0.046 (8.02e-01) | -0.300 (4.36e-01) | -0.553 (4.68e-03) | -0.120 (2.59e-01) | -0.167 (3.08e-01) | 0.158 (3.32e-01) | 0.427 (1.57e-02) | 0.474 (3.08e-02) |
|  | 104910 | Time spent doing moderate physical activity | -0.081 (8.40e-01) | 0.127 (7.72e-01) | -0.170 (5.66e-01) | 0.224 (5.21e-01) | -0.179 (6.06e-01) | -1.026 (9.64e-02) | -0.581 (1.58e-01) | -0.171 (4.71e-01) | -0.332 (2.63e-01) | 0.160 (5.99e-01) | 0.582 (8.89e-02) | 0.785 (3.57e-02) |
| Sedentary Screen Exposure | 1070 | Time spent watching television (TV) | 0.203 (7.25e-05) | -0.125 (2.82e-02) | -0.055 (2.68e-01) | -0.149 (7.36e-03) | 0.058 (2.52e-01) | 0.157 (7.13e-02) | 0.054 (4.04e-01) | -0.039 (3.73e-01) | 0.095 (2.90e-02) | -0.097 (5.70e-02) | -0.294 (5.66e-10) | -0.257 (1.62e-05) |
|  | 1080 | Time spent using computer | -0.187 (7.97e-04) | -0.025 (7.38e-01) | -0.353 (1.78e-22) | 0.311 (7.98e-13) | -0.238 (2.73e-07) | -0.184 (1.10e-01) | -0.193 (9.84e-03) | -0.203 (3.48e-05) | 0.058 (3.45e-01) | -0.300 (2.13e-12) | -0.120 (7.40e-02) | -0.160 (2.55e-02) |
|  | 1110 | Length of mobile phone use | -0.134 (2.66e-02) | -0.046 (5.89e-01) | -0.100 (8.66e-02) | -0.066 (3.76e-01) | -0.139 (9.81e-03) | -0.098 (4.09e-01) | -0.062 (4.31e-01) | 0.082 (6.26e-02) | -0.107 (6.41e-02) | -0.061 (3.01e-01) | -0.053 (4.09e-01) | -0.062 (4.25e-01) |
|  | 1120 | Weekly usage of mobile phone in last 3 months | -0.300 (5.85e-01) | 1.030 (1.01e-01) | 0.458 (3.76e-01) | -0.594 (3.19e-01) | 0.312 (5.15e-01) | -0.947 (2.36e-01) | -0.212 (7.75e-01) | -0.006 (9.87e-01) | 0.315 (4.45e-01) | 0.629 (1.42e-01) | 0.010 (9.84e-01) | 0.990 (8.71e-02) |
| Sleep Related | 1160 | Sleep duration | -0.259 (7.83e-05) | -0.049 (5.75e-01) | 0.002 (9.83e-01) | 0.046 (4.38e-01) | 0.439 (3.22e-22) | -0.266 (2.36e-02) | -0.186 (5.66e-03) | -0.231 (1.58e-08) | 0.172 (6.67e-04) | 0.045 (5.64e-01) | -0.080 (2.94e-01) | 0.091 (2.99e-01) |
|  | 1170 | Getting up in morning | 0.068 (3.79e-01) | 0.056 (4.97e-01) | 0.167 (3.52e-02) | 0.031 (6.47e-01) | -0.144 (3.01e-02) | 0.122 (3.78e-01) | 0.040 (5.75e-01) | -0.396 (2.13e-12) | -0.613 (6.62e-15) | 0.191 (4.23e-02) | 0.278 (1.30e-02) | 0.262 (2.28e-02) |
|  | 1180 | Morning/evening person (chronotype) | -0.012 (8.69e-01) | -0.128 (9.55e-02) | -0.186 (1.98e-04) | 0.057 (3.68e-01) | 0.036 (5.25e-01) | -0.134 (2.94e-01) | -0.046 (4.97e-01) | 0.656 (6.80e-30) | 0.777 (1.73e-31) | -0.196 (5.38e-03) | -0.218 (3.18e-02) | -0.211 (2.34e-02) |
|  | 1190 | Nap during day | -0.083 (4.19e-01) | 0.006 (9.56e-01) | -0.104 (3.42e-01) | -0.036 (5.85e-01) | -0.019 (8.57e-01) | 0.003 (9.85e-01) | 0.200 (9.80e-05) | -0.082 (8.57e-02) | -0.046 (7.47e-01) | -0.219 (8.98e-02) | -0.104 (4.18e-01) | -0.207 (1.79e-01) |
|  | 1200 | Sleeplessness / insomnia | 0.064 (2.87e-01) | 0.127 (1.17e-01) | -0.033 (5.34e-01) | -0.160 (4.17e-03) | -0.168 (3.47e-03) | 0.237 (9.14e-03) | 0.201 (3.55e-03) | 0.089 (7.62e-02) | 0.003 (9.70e-01) | -0.158 (3.09e-04) | -0.138 (1.14e-02) | -0.230 (2.12e-04) |
|  | 20533 | Trouble falling asleep | 0.401 (2.53e-02) | -0.320 (7.49e-02) | 0.198 (3.41e-01) | -0.077 (6.58e-01) | -0.042 (8.57e-01) | -0.029 (9.55e-01) | -0.017 (9.48e-01) | 0.030 (8.25e-01) | 0.056 (8.32e-01) | 0.254 (2.97e-01) | 0.431 (7.91e-02) | 0.780 (5.72e-03) |
|  | 20534 | Sleeping too much | -0.117 (4.98e-01) | 0.113 (6.19e-01) | -0.195 (1.74e-01) | -0.192 (3.00e-01) | 0.224 (1.63e-01) | -0.200 (5.51e-01) | 0.059 (8.20e-01) | 0.327 (5.72e-03) | 0.556 (2.96e-05) | -0.328 (2.41e-02) | -0.263 (2.05e-01) | -0.472 (1.95e-02) |
|  | 1210 | Snoring | -0.059 (4.62e-01) | 0.143 (5.43e-02) | 0.084 (2.78e-01) | 0.108 (6.74e-02) | 0.101 (1.78e-01) | -0.185 (2.61e-01) | -0.067 (4.38e-01) | 0.057 (4.08e-01) | 0.157 (1.07e-01) | 0.074 (4.51e-01) | 0.102 (3.48e-01) | 0.149 (2.13e-01) |
|  | 1220 | Daytime dozing / sleeping (narcolepsy) | -0.028 (7.67e-01) | -0.093 (2.94e-01) | -0.098 (1.90e-01) | -0.116 (8.96e-02) | -0.141 (4.94e-02) | 0.034 (8.35e-01) | 0.091 (2.34e-01) | 0.087 (6.24e-02) | -0.067 (4.45e-01) | -0.235 (5.72e-03) | -0.170 (7.24e-02) | -0.200 (8.03e-02) |
|  | G47 | Diagnoses - main ICD10: G47 Sleep disorders | -0.090 (5.66e-01) | -0.281 (8.89e-02) | -0.230 (2.47e-02) | -0.381 (2.17e-03) | -0.155 (2.24e-01) | 0.096 (7.20e-01) | 0.053 (7.96e-01) | -0.005 (9.70e-01) | -0.111 (3.27e-01) | -0.263 (9.14e-03) | -0.345 (8.68e-03) | -0.245 (1.31e-01) |
|  | G6_SLEEPAPNO | Sleep apnoea | -0.117 (5.11e-01) | -0.385 (4.27e-02) | -0.310 (5.74e-03) | -0.344 (3.25e-02) | -0.137 (3.89e-01) | 0.213 (4.27e-01) | 0.130 (5.23e-01) | -0.004 (9.78e-01) | -0.097 (4.97e-01) | -0.322 (5.94e-03) | -0.420 (9.14e-03) | -0.404 (2.67e-02) |
| Depression | 20421 | Ever felt worried, tense, or anxious for most of a month or longer | -0.062 (5.83e-01) | -0.062 (5.92e-01) | -0.137 (9.09e-02) | -0.005 (9.69e-01) | 0.005 (9.58e-01) | 0.041 (8.02e-01) | -0.114 (2.47e-01) | 0.080 (1.94e-01) | 0.069 (3.77e-01) | -0.161 (4.98e-02) | -0.281 (4.11e-04) | -0.291 (4.12e-03) |
|  | 20425 | Ever worried more than most people would in similar situation | 0.022 (8.52e-01) | 0.142 (1.84e-01) | -0.025 (8.14e-01) | 0.114 (2.26e-01) | -0.073 (4.29e-01) | -0.113 (4.56e-01) | -0.228 (1.32e-02) | 0.020 (7.99e-01) | -0.045 (6.48e-01) | 0.010 (9.33e-01) | 0.014 (9.19e-01) | -0.006 (9.70e-01) |
|  | 20426 | Restless during period of worst anxiety | 0.173 (4.96e-01) | -0.141 (5.94e-01) | 0.236 (2.16e-01) | -0.546 (5.72e-03) | -0.298 (1.67e-01) | 0.358 (3.73e-01) | 0.178 (4.70e-01) | 0.081 (6.47e-01) | -0.045 (8.54e-01) | 0.237 (2.99e-01) | 0.180 (4.59e-01) | -0.057 (8.75e-01) |
|  | 20428 | Professional informed about anxiety | -0.153 (4.03e-01) | -0.033 (9.00e-01) | -0.049 (7.68e-01) | -0.291 (1.29e-01) | 0.553 (7.97e-04) | -0.839 (5.19e-03) | -0.401 (7.74e-02) | -0.084 (5.23e-01) | 0.218 (1.58e-01) | -0.128 (4.33e-01) | -0.115 (5.74e-01) | -0.293 (1.55e-01) |
|  | 20448 | Professional informed about depression | -0.260 (2.70e-02) | -0.187 (1.79e-01) | -0.250 (6.57e-03) | 0.210 (1.15e-01) | -0.036 (7.96e-01) | -0.178 (4.59e-01) | -0.169 (2.42e-01) | -0.050 (5.98e-01) | -0.008 (9.56e-01) | -0.145 (2.03e-01) | -0.110 (4.24e-01) | -0.032 (8.75e-01) |
|  | 20126_2 | Bipolar and major depression status: Bipolar II Disorder | 0.164 (5.45e-01) | 0.507 (1.32e-01) | 0.038 (8.75e-01) | -0.182 (5.25e-01) | 0.133 (6.19e-01) | -1.186 (3.55e-03) | -0.227 (5.36e-01) | 0.191 (3.85e-01) | 0.284 (1.29e-01) | 0.041 (8.75e-01) | 0.197 (4.59e-01) | 0.327 (2.82e-01) |
|  | KRA_PSY_ANXIETY | Anxiety disorders | 0.203 (5.02e-01) | 0.117 (7.94e-01) | 0.170 (5.14e-01) | 0.088 (7.77e-01) | 0.711 (5.19e-03) | 0.501 (3.01e-01) | -0.533 (6.63e-02) | -0.253 (2.40e-01) | -0.078 (7.94e-01) | 0.303 (2.99e-01) | -0.008 (9.83e-01) | 0.599 (1.26e-01) |
|  | KRA_PSY_ANYMENTAL | Any mental disorder | 0.150 (2.56e-01) | 0.104 (5.60e-01) | 0.036 (7.65e-01) | -0.167 (1.60e-01) | 0.038 (7.93e-01) | 0.089 (7.93e-01) | -0.198 (1.92e-01) | -0.094 (3.77e-01) | 0.046 (7.22e-01) | 0.014 (9.23e-01) | -0.251 (1.25e-01) | -0.153 (4.46e-01) |
|  | F5_DEPRESSIO | Depression | -0.042 (8.35e-01) | 0.081 (7.15e-01) | 0.034 (8.43e-01) | 0.111 (5.04e-01) | 0.028 (8.72e-01) | 0.357 (1.73e-01) | 0.030 (8.87e-01) | 0.044 (7.47e-01) | 0.076 (5.85e-01) | -0.098 (4.91e-01) | -0.360 (1.40e-02) | -0.249 (2.06e-01) |
|  | F5_SCHIZO | Schizophrenia, schizotypal and delusional disorders | -0.327 (2.94e-01) | -0.243 (5.45e-01) | 0.075 (7.96e-01) | -0.420 (1.40e-01) | -0.161 (5.85e-01) | 0.484 (2.71e-01) | 0.455 (2.59e-01) | 0.281 (1.79e-01) | 0.133 (6.34e-01) | -0.064 (8.34e-01) | -0.372 (2.51e-01) | -0.142 (7.38e-01) |
| BMI and Diet | 21001_irnt | Body mass index (BMI) | 0.027 (6.24e-01) | -0.183 (8.99e-05) | -0.166 (1.93e-07) | -0.216 (2.63e-07) | -0.150 (2.56e-05) | 0.026 (7.71e-01) | 0.052 (4.10e-01) | 0.051 (1.59e-01) | -0.016 (7.41e-01) | -0.233 (9.15e-13) | -0.318 (9.49e-20) | -0.349 (8.87e-14) |
|  | 21002_irnt | Weight | 0.049 (3.01e-01) | -0.175 (1.70e-05) | -0.179 (5.10e-09) | -0.166 (6.40e-05) | -0.120 (4.76e-04) | -0.062 (4.34e-01) | 0.029 (6.22e-01) | 0.015 (7.06e-01) | -0.026 (5.07e-01) | -0.273 (9.68e-20) | -0.320 (1.42e-17) | -0.374 (1.30e-17) |
|  | 1478 | Salt added to food | 0.036 (5.31e-01) | -0.137 (3.87e-02) | -0.077 (1.11e-01) | -0.072 (2.69e-01) | -0.072 (1.60e-01) | 0.119 (1.32e-01) | 0.071 (2.51e-01) | 0.002 (9.71e-01) | 0.010 (8.53e-01) | -0.051 (2.84e-01) | -0.167 (1.81e-03) | -0.094 (1.60e-01) |
|  | 1498 | Coffee intake | -0.051 (5.00e-01) | 0.005 (9.62e-01) | -0.022 (7.24e-01) | -0.032 (6.87e-01) | -0.110 (6.63e-02) | -0.253 (7.90e-02) | 0.048 (5.76e-01) | 0.009 (8.93e-01) | -0.054 (3.03e-01) | -0.002 (9.76e-01) | 0.121 (5.50e-02) | 0.025 (7.98e-01) |
|  | 100001_irnt | Food weight | 0.104 (3.58e-01) | 0.043 (7.97e-01) | 0.171 (7.66e-02) | -0.100 (4.05e-01) | -0.248 (1.48e-02) | -0.180 (4.28e-01) | -0.242 (9.55e-02) | 0.163 (1.07e-01) | 0.010 (9.43e-01) | 0.177 (8.59e-02) | 0.212 (1.24e-01) | 0.383 (4.26e-03) |
|  | 100002_irnt | Energy | 0.250 (3.87e-02) | 0.313 (6.92e-02) | 0.269 (9.81e-03) | 0.038 (8.16e-01) | 0.010 (9.48e-01) | 0.001 (9.96e-01) | 0.031 (8.77e-01) | 0.167 (6.69e-02) | 0.188 (1.34e-01) | 0.144 (2.36e-01) | 0.229 (1.22e-01) | 0.216 (2.13e-01) |
|  | 100003_irnt | Protein | 0.342 (1.05e-02) | 0.190 (3.44e-01) | 0.272 (2.69e-02) | -0.074 (6.48e-01) | -0.014 (9.33e-01) | 0.039 (8.88e-01) | 0.036 (8.88e-01) | 0.213 (3.87e-02) | 0.111 (4.50e-01) | 0.106 (4.38e-01) | 0.165 (3.02e-01) | 0.089 (6.56e-01) |
|  | 100004_irnt | Fat | 0.244 (1.34e-01) | 0.316 (1.12e-01) | 0.230 (1.02e-01) | 0.028 (8.89e-01) | -0.149 (3.67e-01) | -0.030 (9.31e-01) | 0.110 (5.85e-01) | 0.238 (3.05e-02) | 0.161 (3.41e-01) | 0.016 (9.36e-01) | 0.150 (4.13e-01) | 0.114 (5.97e-01) |
|  | 100005_irnt | Carbohydrate | 0.254 (3.01e-02) | 0.269 (8.06e-02) | 0.296 (3.49e-03) | 0.047 (7.56e-01) | -0.120 (3.36e-01) | -0.296 (1.99e-01) | -0.126 (3.95e-01) | 0.040 (6.91e-01) | 0.094 (4.90e-01) | 0.205 (1.08e-01) | 0.346 (3.43e-02) | 0.313 (9.45e-02) |
| Alcohol  Consumption | 20404 | Ever physically dependent on alcohol | 3.541 (2.54e-03) | -2.080 (1.69e-01) | -0.158 (8.82e-01) | -0.171 (9.11e-01) | 1.511 (2.35e-01) | 0.768 (7.47e-01) | -0.628 (7.62e-01) | 0.344 (7.63e-01) | -0.009 (9.94e-01) | -0.116 (9.19e-01) | -0.070 (9.66e-01) | -1.121 (4.89e-01) |
|  | 20406 | Ever addicted to alcohol | 0.086 (7.06e-01) | 0.142 (5.31e-01) | 0.038 (8.54e-01) | 0.015 (9.46e-01) | 0.140 (4.02e-01) | 0.200 (5.36e-01) | 0.267 (1.57e-01) | -0.218 (4.77e-02) | 0.114 (5.25e-01) | 0.166 (3.58e-01) | 0.023 (9.26e-01) | 0.118 (6.58e-01) |
|  | 20117_1 | Alcohol drinker status: Previous | 0.232 (1.95e-02) | 0.005 (9.72e-01) | 0.044 (6.18e-01) | -0.148 (1.56e-01) | -0.114 (1.84e-01) | 0.108 (5.36e-01) | 0.195 (8.71e-02) | -0.109 (1.28e-01) | -0.089 (2.82e-01) | -0.029 (7.67e-01) | -0.074 (4.97e-01) | -0.158 (2.33e-01) |
|  | 20117_2 | Alcohol drinker status: Current | -0.145 (5.18e-02) | -0.041 (7.05e-01) | -0.054 (4.36e-01) | 0.202 (5.72e-03) | 0.103 (1.87e-01) | -0.063 (6.10e-01) | -0.099 (2.56e-01) | 0.088 (1.14e-01) | 0.109 (1.25e-01) | 0.061 (4.11e-01) | -0.010 (9.22e-01) | 0.086 (3.65e-01) |
| Shift Workers | 22620_0 | Job involved shift work: No | -0.018 (9.70e-01) | 0.418 (3.12e-01) | 0.452 (1.59e-01) | 0.155 (6.93e-01) | 0.253 (4.97e-01) | 0.794 (2.50e-01) | 0.475 (2.75e-01) | -0.100 (7.52e-01) | 0.113 (8.01e-01) | 0.325 (4.27e-01) | 0.144 (7.63e-01) | -0.103 (8.75e-01) |
|  | 22620_1 | Job involved shift work: Yes | 0.114 (3.43e-01) | 0.078 (6.32e-01) | 0.051 (6.45e-01) | -0.383 (5.87e-04) | -0.087 (4.93e-01) | 0.178 (4.01e-01) | 0.214 (1.31e-01) | 0.059 (5.93e-01) | -0.034 (7.92e-01) | -0.034 (7.91e-01) | -0.113 (4.32e-01) | -0.233 (1.24e-01) |
| Other Diseases | 20002_1112 | Non-cancer illness code, self-reported: chronic obstructive airways disease/copd | 0.109 (4.97e-01) | -0.021 (9.31e-01) | 0.188 (1.79e-01) | -0.278 (9.13e-02) | -0.144 (3.20e-01) | 0.497 (7.40e-02) | 0.454 (1.03e-02) | -0.050 (6.86e-01) | -0.053 (7.18e-01) | 0.099 (5.25e-01) | -0.190 (2.56e-01) | -0.169 (4.71e-01) |
|  | 20002_1398 | Non-cancer illness code, self-reported: pneumonia | 0.359 (7.60e-02) | -0.263 (3.03e-01) | -0.088 (6.16e-01) | -0.025 (9.28e-01) | 0.080 (6.93e-01) | 0.132 (6.97e-01) | 0.370 (1.10e-01) | 0.350 (4.36e-02) | 0.195 (3.22e-01) | -0.168 (3.96e-01) | -0.031 (9.20e-01) | -0.228 (4.42e-01) |
|  | 20002_1111 | Non-cancer illness code, self-reported: asthma | 0.107 (1.35e-01) | -0.004 (9.67e-01) | 0.048 (3.99e-01) | -0.153 (1.84e-03) | 0.071 (4.33e-01) | 0.054 (5.75e-01) | 0.168 (1.61e-02) | -0.023 (6.93e-01) | -0.013 (8.29e-01) | -0.005 (9.39e-01) | -0.147 (2.68e-03) | -0.188 (1.48e-02) |
|  | ASTHMA_HOSPITAL1 | Asthma, hospital admissions 1 | 0.115 (4.80e-01) | -0.080 (6.49e-01) | 0.217 (4.51e-02) | -0.387 (5.72e-03) | 0.451 (2.43e-03) | 0.436 (8.44e-02) | 0.518 (7.41e-03) | -0.156 (1.95e-01) | 0.109 (3.74e-01) | 0.241 (4.75e-02) | -0.323 (2.00e-02) | -0.265 (1.50e-01) |
|  | ASTHMA_CHILD | Childhood asthma (age<16) | 0.103 (5.31e-01) | -0.069 (7.00e-01) | 0.213 (4.75e-02) | -0.388 (5.72e-03) | 0.442 (3.14e-03) | 0.441 (8.52e-02) | 0.512 (9.14e-03) | -0.158 (1.84e-01) | 0.115 (3.42e-01) | 0.234 (5.01e-02) | -0.326 (1.79e-02) | -0.264 (1.52e-01) |
|  | 20002_1446 | Non-cancer illness code, self-reported: anaemia | 0.675 (9.68e-03) | -0.078 (8.40e-01) | 0.043 (8.75e-01) | -0.181 (5.80e-01) | 0.555 (3.14e-02) | -0.681 (7.98e-02) | 0.085 (8.12e-01) | -0.211 (3.38e-01) | 0.102 (7.17e-01) | -0.137 (5.85e-01) | -0.227 (4.69e-01) | 0.030 (9.45e-01) |
|  | D3_ANAEMIA_IRONDEF | Iron deficiency anaemia | 0.404 (6.80e-03) | -0.174 (3.86e-01) | 0.231 (5.01e-02) | -0.205 (2.14e-01) | -0.008 (9.67e-01) | 0.203 (4.37e-01) | 0.208 (2.56e-01) | -0.008 (9.62e-01) | 0.053 (6.66e-01) | 0.044 (7.67e-01) | -0.158 (3.76e-01) | -0.384 (5.01e-02) |
|  | D3_ANAEMIANAS | Other and unspecified anaemias | 0.080 (7.03e-01) | -0.300 (1.79e-01) | -0.259 (1.34e-01) | -0.250 (1.64e-01) | -0.021 (9.36e-01) | -0.220 (5.89e-01) | 0.405 (1.13e-01) | 0.015 (9.39e-01) | 0.122 (4.58e-01) | -0.202 (2.21e-01) | -0.222 (2.20e-01) | -0.471 (3.30e-02) |
|  | D3_OTHERANAEMIA | Other anaemias | 0.080 (7.05e-01) | -0.304 (1.73e-01) | -0.259 (1.34e-01) | -0.236 (1.94e-01) | -0.022 (9.33e-01) | -0.226 (5.80e-01) | 0.406 (1.12e-01) | 0.016 (9.38e-01) | 0.123 (4.52e-01) | -0.206 (2.13e-01) | -0.220 (2.27e-01) | -0.475 (3.20e-02) |
|  | J10_EXTERLUNG | Lung diseases due to external agents | -0.409 (3.10e-01) | -0.390 (3.68e-01) | 0.345 (1.95e-01) | 0.032 (9.41e-01) | -0.150 (6.87e-01) | 0.100 (9.04e-01) | 0.515 (2.78e-01) | 0.108 (7.05e-01) | -0.322 (2.73e-01) | 0.354 (2.49e-01) | 0.782 (3.18e-02) | 0.110 (8.35e-01) |
