# Supplementary material for: Automated feature extraction from population wearable device data identified novel loci associated with sleep and circadian rhythms
Source: PLoS Genet. 2020 Oct 19;16(10):e1009089. doi: 10.1371/journal.pgen.1009089 (PMC7595622; doi:10.1371/journal.pgen.1009089)
Supplement: S4 Table — (DOCX) [file pgen.1009089.s006.docx]

S4 Table. Single-tissue gene-trait association test results from tissue enrichment analysis using UTMOST.

| Phenotype | Tissue | GENE | zscore | pvalue |
| --- | --- | --- | --- | --- |
| Mean Activity Levels During Sleep | Esophagus_Muscularis | BRSK1 | 4.63 | 3.70E-06 |
| Mean Activity Levels During Sleep | Prostate | BRSK1 | 4.80 | 1.57E-06 |
| Mean Activity Levels During Sleep | Skin_Not_Sun_Exposed_Suprapubic | BRSK1 | 4.71 | 2.52E-06 |
| Mean Activity Levels During Sleep | Brain_Frontal_Cortex_BA9 | CEP70 | -4.59 | 4.46E-06 |
| Mean Activity Levels During Sleep | Esophagus_Gastroesophageal_Junction | CEP70 | -4.68 | 2.84E-06 |
| Mean Activity Levels During Sleep | Brain_Hypothalamus | CEP70 | -4.61 | 4.09E-06 |
| Mean Activity Levels During Sleep | Lung | CEP70 | -4.63 | 3.73E-06 |
| Mean Activity Levels During Sleep | Heart_Left_Ventricle | KATNAL2 | 4.64 | 3.41E-06 |
| Mean Activity Levels During Sleep | Brain_Nucleus_accumbens_basal_ganglia | KCNH7 | -5.01 | 5.35E-07 |
| Mean Activity Levels During Sleep | Adipose_Subcutaneous | L3MBTL2 | -4.75 | 2.07E-06 |
| Mean Activity Levels During Sleep | Whole_Blood | L3MBTL2 | -4.72 | 2.38E-06 |
| Mean Activity Levels During Sleep | Brain_Hippocampus | PLA2G4B | -4.84 | 1.31E-06 |
| Mean Activity Levels During Wake | Brain_Putamen_basal_ganglia | ARID3B | 4.81 | 1.54E-06 |
| Mean Activity Levels During Wake | Heart_Left_Ventricle | BEAN1 | 4.70 | 2.57E-06 |
| Mean Activity Levels During Wake | Adipose_Subcutaneous | GIPC2 | -4.72 | 2.32E-06 |
| Mean Activity Levels During Wake | Heart_Left_Ventricle | GIPC2 | 4.83 | 1.35E-06 |
| Mean Activity Levels During Wake | Breast_Mammary_Tissue | GIPC2 | -4.74 | 2.12E-06 |
| Mean Activity Levels During Wake | Adipose_Subcutaneous | L3MBTL2 | -5.39 | 7.05E-08 |
| Mean Activity Levels During Wake | Muscle_Skeletal | L3MBTL2 | -5.62 | 1.92E-08 |
| Mean Activity Levels During Wake | Liver | MBD2 | 4.78 | 1.71E-06 |
| Mean Activity Levels During Wake | Brain_Anterior_cingulate_cortex_BA24 | PRUNE | -4.67 | 3.02E-06 |
| Mean Activity Levels During Wake | Whole_Blood | SEC11C | -4.70 | 2.63E-06 |
| Mean Activity Levels During Wake | Brain_Anterior_cingulate_cortex_BA24 | SOD3 | -4.75 | 2.06E-06 |
| Mean Activity Levels During Wake | Muscle_Skeletal | SPARC | -5.03 | 4.86E-07 |
| Mean Activity Levels During Wake | Adrenal_Gland | TRAF3 | 4.87 | 1.14E-06 |
| Mean Activity Levels During Wake | Artery_Aorta | TRAF3 | 4.87 | 1.12E-06 |
| Mean Activity Levels During Wake | Brain_Cerebellar_Hemisphere | TRAF3 | 4.67 | 3.01E-06 |
| Mean Activity Levels During Wake | Brain_Frontal_Cortex_BA9 | TRAF3 | 5.11 | 3.25E-07 |
| Mean Activity Levels During Wake | Esophagus_Gastroesophageal_Junction | TRAF3 | 4.77 | 1.85E-06 |
| Mean Activity Levels During Wake | Esophagus_Muscularis | TRAF3 | 4.86 | 1.18E-06 |
| Mean Activity Levels During Wake | Liver | TRAF3 | 4.88 | 1.07E-06 |
| Sleep Duration | Vagina | ADAMTS7 | 4.80 | 1.63E-06 |
| Sleep Duration | Adipose_Subcutaneous | GLTP | 4.66 | 3.13E-06 |
| Sleep Duration | Adipose_Visceral_Omentum | GLTP | -4.76 | 1.90E-06 |
| Sleep Duration | Adrenal_Gland | GLTP | -4.71 | 2.42E-06 |
| Sleep Duration | Artery_Aorta | GLTP | 4.76 | 1.97E-06 |
| Sleep Duration | Artery_Coronary | GLTP | 4.74 | 2.11E-06 |
| Sleep Duration | Artery_Tibial | GLTP | -4.74 | 2.12E-06 |
| Sleep Duration | Brain_Anterior_cingulate_cortex_BA24 | GLTP | -4.75 | 2.07E-06 |
| Sleep Duration | Brain_Caudate_basal_ganglia | GLTP | -4.73 | 2.25E-06 |
| Sleep Duration | Brain_Cerebellar_Hemisphere | GLTP | -4.76 | 1.95E-06 |
| Sleep Duration | Brain_Cortex | GLTP | -4.75 | 2.01E-06 |
| Sleep Duration | Brain_Hippocampus | GLTP | -4.75 | 1.99E-06 |
| Sleep Duration | Brain_Hypothalamus | GLTP | -4.76 | 1.92E-06 |
| Sleep Duration | Brain_Nucleus_accumbens_basal_ganglia | GLTP | -4.70 | 2.55E-06 |
| Sleep Duration | Brain_Putamen_basal_ganglia | GLTP | -4.69 | 2.77E-06 |
| Sleep Duration | Cells_EBV-transformed_lymphocytes | GLTP | 4.61 | 3.97E-06 |
| Sleep Duration | Cells_Transformed_fibroblasts | GLTP | 4.75 | 1.99E-06 |
| Sleep Duration | Colon_Sigmoid | GLTP | 4.73 | 2.26E-06 |
| Sleep Duration | Colon_Transverse | GLTP | -4.76 | 1.94E-06 |
| Sleep Duration | Esophagus_Gastroesophageal_Junction | GLTP | -4.75 | 2.00E-06 |
| Sleep Duration | Esophagus_Mucosa | GLTP | 4.73 | 2.29E-06 |
| Sleep Duration | Esophagus_Muscularis | GLTP | 4.65 | 3.28E-06 |
| Sleep Duration | Heart_Atrial_Appendage | GLTP | 4.63 | 3.68E-06 |
| Sleep Duration | Heart_Left_Ventricle | GLTP | -4.74 | 2.15E-06 |
| Sleep Duration | Liver | GLTP | 4.73 | 2.24E-06 |
| Sleep Duration | Lung | GLTP | -4.75 | 2.01E-06 |
| Sleep Duration | Vagina | GLTP | -4.75 | 1.98E-06 |
| Sleep Duration | Muscle_Skeletal | GLTP | -4.73 | 2.27E-06 |
| Sleep Duration | Nerve_Tibial | GLTP | -4.76 | 1.89E-06 |
| Sleep Duration | Ovary | GLTP | -4.75 | 2.02E-06 |
| Sleep Duration | Pancreas | GLTP | -4.76 | 1.98E-06 |
| Sleep Duration | Prostate | GLTP | 4.73 | 2.26E-06 |
| Sleep Duration | Skin_Not_Sun_Exposed_Suprapubic | GLTP | -4.75 | 2.03E-06 |
| Sleep Duration | Small_Intestine_Terminal_Ileum | GLTP | 4.77 | 1.88E-06 |
| Sleep Duration | Spleen | GLTP | 4.76 | 1.94E-06 |
| Sleep Duration | Stomach | GLTP | -4.77 | 1.89E-06 |
| Sleep Duration | Testis | GLTP | 4.75 | 2.06E-06 |
| Sleep Duration | Uterus | GLTP | 4.73 | 2.28E-06 |
| Sleep Duration | Whole_Blood | GLTP | 4.67 | 2.94E-06 |
| Sleep Duration | Lung | KIFC3 | 4.65 | 3.28E-06 |
| Sleep Start | Artery_Coronary | ABCD2 | 4.71 | 2.53E-06 |
| Sleep Start | Brain_Anterior_cingulate_cortex_BA24 | ABCD2 | -4.61 | 3.94E-06 |
| Sleep Start | Adipose_Visceral_Omentum | ABCD2 | 4.76 | 1.94E-06 |
| Sleep Start | Brain_Cortex | ABCD2 | 4.62 | 3.90E-06 |
| Sleep Start | Brain_Cerebellum | ABCD2 | -4.78 | 1.79E-06 |
| Sleep Start | Colon_Sigmoid | ABCD2 | 4.61 | 4.11E-06 |
| Sleep Start | Adipose_Subcutaneous | ABCD2 | 4.75 | 2.03E-06 |
| Sleep Start | Colon_Transverse | ABCD2 | 4.67 | 3.02E-06 |
| Sleep Start | Whole_Blood | ABCD2 | -4.58 | 4.55E-06 |
| Sleep Start | Small_Intestine_Terminal_Ileum | ALG10B | 4.94 | 7.99E-07 |
| Sleep Start | Whole_Blood | ALG10B | -4.75 | 1.99E-06 |
| Sleep Start | Adipose_Subcutaneous | CCDC181 | -4.81 | 1.49E-06 |
| Sleep Start | Lung | CCDC87 | -4.63 | 3.66E-06 |
| Sleep Start | Brain_Anterior_cingulate_cortex_BA24 | CEP128 | -4.62 | 3.85E-06 |
| Sleep Start | Prostate | FSIP2 | -4.65 | 3.28E-06 |
| Sleep Start | Nerve_Tibial | KIAA0391 | -4.65 | 3.31E-06 |
| Sleep Start | Brain_Cerebellum | LIMS1 | 4.90 | 9.76E-07 |
| Sleep Start | Brain_Nucleus_accumbens_basal_ganglia | LIMS1 | 5.02 | 5.18E-07 |
| Sleep Start | Testis | LIMS1 | -4.81 | 1.50E-06 |
| Sleep Start | Brain_Frontal_Cortex_BA9 | LIMS1 | 4.95 | 7.61E-07 |
| Sleep Start | Esophagus_Mucosa | LRFN4 | 4.80 | 1.62E-06 |
| Sleep Start | Brain_Anterior_cingulate_cortex_BA24 | LRFN4 | 4.63 | 3.59E-06 |
| Sleep Start | Adrenal_Gland | LRFN4 | 4.65 | 3.26E-06 |
| Sleep Start | Artery_Coronary | LRFN4 | 4.62 | 3.76E-06 |
| Sleep Start | Colon_Sigmoid | LRFN4 | 4.64 | 3.55E-06 |
| Sleep Start | Ovary | LRFN4 | 4.79 | 1.65E-06 |
| Sleep Start | Uterus | MYO18A | -5.05 | 4.39E-07 |
| Sleep Start | Brain_Cortex | STX17 | -4.65 | 3.37E-06 |
| Sleep Start | Colon_Transverse | TREH | 7.23 | 4.68E-13 |
| Sleep Start | Stomach | TREH | 5.76 | 8.54E-09 |
| Sleep Start | Esophagus_Mucosa | TREH | 5.53 | 3.15E-08 |
| Sleep End | Whole_Blood | HILPDA | -4.66 | 3.19E-06 |
| Sleep End | Brain_Cerebellum | JARID2 | -5.13 | 2.95E-07 |
| Sleep End | Artery_Coronary | SEPN1 | -4.69 | 2.73E-06 |
| Sleep End | Brain_Cortex | SEPN1 | -4.61 | 3.94E-06 |
| 1-day Periodicity | Brain_Cerebellar_Hemisphere | C1orf162 | 4.91 | 9.06E-07 |
| 1-day Periodicity | Liver | C1orf162 | -4.94 | 7.97E-07 |
| 1-day Periodicity | Adipose_Subcutaneous | L3MBTL2 | -5.03 | 4.81E-07 |
| 1-day Periodicity | Breast_Mammary_Tissue | L3MBTL2 | -4.77 | 1.82E-06 |
| 1-day Periodicity | Muscle_Skeletal | L3MBTL2 | -4.83 | 1.38E-06 |
| 1-day Periodicity | Muscle_Skeletal | SPARC | -5.28 | 1.30E-07 |
| 1-day Periodicity | Artery_Tibial | ZFAND2B | 4.73 | 2.26E-06 |
| 1-day Periodicity | Cells_EBV-transformed_lymphocytes | ZNF79 | -4.63 | 3.72E-06 |
| 1/2-day Periodicity | Esophagus_Mucosa | CPZ | -4.78 | 1.79E-06 |
| 1/2-day Periodicity | Pancreas | DCTPP1 | 4.72 | 2.38E-06 |
| 1/2-day Periodicity | Esophagus_Mucosa | PCDH9 | 4.61 | 3.98E-06 |
| 1/2-day Periodicity | Muscle_Skeletal | PCDH9 | 4.61 | 3.98E-06 |
| 1/2-day Periodicity | Prostate | PCDH9 | -4.61 | 3.98E-06 |
| 1/2-day Periodicity | Vagina | PCDH9 | 4.61 | 3.98E-06 |
| 1/2-day Periodicity | Brain_Hypothalamus | PRSS36 | 4.65 | 3.39E-06 |
| 1/2-day Periodicity | Brain_Putamen_basal_ganglia | STARD3 | -4.89 | 1.02E-06 |
| 1/3-day Periodicity | Brain_Anterior_cingulate_cortex_BA24 | PDE7B | 4.63 | 3.59E-06 |
| 1/3-day Periodicity | Muscle_Skeletal | STATH | 4.97 | 6.74E-07 |
