# Supplementary material for: Automated feature extraction from population wearable device data identified novel loci associated with sleep and circadian rhythms
Source: PLoS Genet. 2020 Oct 19;16(10):e1009089. doi: 10.1371/journal.pgen.1009089 (PMC7595622; doi:10.1371/journal.pgen.1009089)
Supplement: S5 Table — (DOCX) [file pgen.1009089.s007.docx]

S5 Table. Cross-tissue gene-trait association test results from tissue enrichment analysis using UTMOST.

| Phenotype | gene | test_score | p_value |
| --- | --- | --- | --- |
| Mean Activity Levels During Sleep | CA7 | 13.14 | 2.53E-06 |
| Mean Activity Levels During Sleep | LYVE1 | 13.80 | 2.48E-06 |
| Mean Activity Levels During Sleep | PIAS2 | 15.82 | 6.13E-08 |
| Activity Variability During Sleep | FTL | 21.53 | 1.15E-09 |
| Mean Activity Levels During Wake | CA7 | 23.03 | 1.28E-10 |
| Mean Activity Levels During Wake | L3MBTL2 | 12.32 | 2.16E-06 |
| Mean Activity Levels During Wake | TRAF3 | 14.66 | 3.35E-07 |
| Activity Variability During Wake | CA7 | 27.10 | 5.19E-12 |
| Activity Variability During Wake | DUSP15 | 12.25 | 1.64E-06 |
| Activity Variability During Wake | ELMOD2 | 43.91 | 2.91E-11 |
| Activity Variability During Wake | POM121L7 | 13.40 | 1.96E-06 |
| Activity Variability During Wake | SLC7A4 | 12.85 | 2.20E-06 |
| Sleep Duration | DYNC1LI1 | 17.77 | 5.76E-08 |
| Sleep Duration | ELMOD2 | 59.88 | 2.91E-11 |
| Sleep Duration | GDPD5 | 15.22 | 1.56E-07 |
| Sleep Duration | GLTP | 14.21 | 1.11E-06 |
| Sleep Start | ABCD2 | 150.41 | 8.38E-07 |
| Sleep Start | DCLK2 | 13.67 | 7.72E-07 |
| Sleep Start | DYNC1LI1 | 49.66 | 2.91E-11 |
| Sleep Start | KIDINS220 | 17.58 | 1.40E-08 |
| Sleep Start | PPP2R2C | 13.14 | 1.16E-06 |
| Sleep Start | TREH | 23.76 | 2.01E-11 |
| Sleep End | ELMOD2 | 54.35 | 2.91E-11 |
| Sleep End | GRIA1 | 13.33 | 1.44E-06 |
| Sleep End | VCAM1 | 35.73 | 2.91E-11 |
| 1-day Periodicity | C1orf162 | 12.84 | 1.90E-06 |
| 1/2-day Periodicity | ALS2 | 11.95 | 2.28E-06 |
| 1/2-day Periodicity | ALS2CR11 | 19.56 | 1.64E-09 |
| 1/2-day Periodicity | CPZ | 14.81 | 2.12E-07 |
| 1/2-day Periodicity | DPP9 | 16.21 | 2.84E-08 |
| 1/2-day Periodicity | DYNC1LI1 | 21.36 | 1.63E-09 |
| 1/2-day Periodicity | ELMOD2 | 20.29 | 4.83E-09 |
| 1/3-day Periodicity | DYNC1LI1 | 27.16 | 3.36E-11 |
| 1/3-day Periodicity | ELMOD2 | 23.54 | 1.98E-10 |
