# Supplementary material for: Automated feature extraction from population wearable device data identified novel loci associated with sleep and circadian rhythms
Source: PLoS Genet. 2020 Oct 19;16(10):e1009089. doi: 10.1371/journal.pgen.1009089 (PMC7595622; doi:10.1371/journal.pgen.1009089)
Supplement: S6 Table — (DOCX) [file pgen.1009089.s008.docx]

S6 Table. Two-sample Mendelian Randomization analysis for the strength of circadian rhythm using GWAS summary statistics from the GIANT study.

| Exposure | Outcome | Outcome units | Method | N SNPs | beta | SE | P Value |
| --- | --- | --- | --- | --- | --- | --- | --- |
| Strength of Circadian Rhythm | Body mass index (BMI) | SD (Kg/m2) | Inverse variance weighted | 11 | -0.00050 | 0.00014 | 3.42E-04 |
| Strength of Circadian Rhythm | Body mass index (BMI) | SD (Kg/m2) | Weighted median | 11 | -0.00067 | 0.00010 | 1.81E-11 |
| Strength of Circadian Rhythm | Body mass index (BMI) | SD (Kg/m2) | Weighted mode | 11 | -0.00071 | 0.00011 | 7.93E-05 |
| Strength of Circadian Rhythm | Body mass index (BMI) | SD (Kg/m2) | MR Egger | 11 | -0.00152 | 0.00103 | 0.174 |
